# Human Health and Ecological Risk Assessment of 16 Polycyclic Aromatic Hydrocarbons in Drinking Source Water from a Large Mixed-Use Reservoir

**DOI:** 10.3390/ijerph121113956

**Published:** 2015-10-30

**Authors:** Caiyun Sun, Jiquan Zhang, Qiyun Ma, Yanan Chen

**Affiliations:** Department of Environment, Institute of Natural Disaster Research, Northeast Normal University, Changchun 130024, China; E-Mails: suncaiyun@hotmail.com (C.S.); mqy199312@gmail.com (Q.M.); chenyn061@nenu.edu.cn (Y.C.)

**Keywords:** water, polycyclic aromatic hydrocarbons, ecological risk assessment, human health risk assessment, PAH sources

## Abstract

Reservoirs play an important role in living water supply and irrigation of farmlands, thus the water quality is closely related to public health. However, studies regarding human health and ecological risk assessment of polycyclic aromatic hydrocarbons (PAHs) in the waters of reservoirs are very few. In this study, Shitou Koumen Reservoir which supplies drinking water to 8 million people was investigated. Sixteen priority PAHs were analyzed in a total of 12 water samples. In terms of the individual PAHs, the average concentration of Fla, which was 5.66 × 10^−1^ μg/L, was the highest, while dibenz(a,h)anthracene which was undetected in any of the water samples was the lowest. Among three PAH compositional patterns, the concentration of low-molecular-weight and 4-ring PAHs was dominant, accounting for 94%, and the concentration of the total of 16 PAHs was elevated in constructed-wetland and fish-farming areas. According to the calculated risk quotients, little or no adverse effects were posed by individual and complex PAHs in the water on the aquatic ecosystem. In addition, the results of hazard quotients for non-carcinogenic risk also showed little or no negative impacts on the health of local residents. However, it could be concluded from the carcinogenic risk results that chrysene and complex PAHs in water might pose a potential carcinogenic risk to local residents. Moreover, the possible sources of PAHs were identified as oil spills and vehicular emissions, as well as the burning of biomass and coal.

## 1. Introduction

Reservoirs play an important role in the drinking water supply and irrigation of farmlands, thus the water quality is closely related to the public health. However, available information about the risk assessment of reservoir water quality on public health is very limited.

Located in Jilin Province of China, the Shitou Koumen Reservoir (43°58′ N, 125°45′ E) is the primary water source for farmland irrigation and the living water supply for eight million local residents. It is also utilized in fish farming, the production of which hits 130 tons. As Shitou Koumen Reservoir supplies the living water for 8 million local residents and the agricultural food and fish products are also consumed by local residents, the water quality in this reservoir can greatly influence the health of local residents. The Shitou Koumen Reservoir is fed by the Yinma River, Shuangyang River and Chalu River where the pollution is increasingly fierce with the rapid development of industry, agriculture and urban construction over the last 20 years. The great environmental pressure on Shitou Koumen Reservoir may also be caused by oil-related activities of large-scale oil refineries in cities along the rivers.

Sewage of industrial plants, agricultural runoffs, aquaculture industry and oil-related activities are all possible sources of PAHs [1,2,3]. PAHs can adversely affect not only human health through drinking water but also sensitive species of organisms [1]. A great amount of PAHs may enter the water bodies, and accumulate, bio-accumulate or bio-magnify in food chains [4,5]. The negative impacts on human health and the ecosystem due to their teratogenic, carcinogenic and mutagenic characteristics may be caused by the accumulation of high levels of PAHs in environmental compartments [6]. Meanwhile, the negative impacts may be even greater in the aquatic environment [7]. Although the water quality of Shitou Koumen Reservoir is closely related to the health of eight million local residents, to our best knowledge, there are few or even no studies concerning the risk assessment of PAHs in the waters of Shitou Koumen Reservoir.

This study focused on human health and ecological risk assessment of PAHs in the waters of Shitou Koumen Reservoir. The traditional method used for evaluating human health risk caused by the exposure of pollutants is just by comparing the monitoring concentration with some maximum allowable concentration, which is not sufficient however [8]. In this study, hazard quotients (HQs) for non-carcinogenic risk and carcinogenic risk which can assess the risk from a lifetime intake of pollutants [9] were applied to the assessment of human health risk. At the same time, risk quotient (RQs) can be used to assess the potential risk to aquatic ecosystems [10]. In order to carry out the ecological risk assessment more accurately, RQs under best and worst cases which could usually judge the risk level independently were adopted [11]. 

Among a great number of PAH species, sixteen PAHs recommended as priority pollutants by the United States Environmental Protection Agency (USEPA) [12] were investigated. The objectives of this study were mainly to study the distribution characteristics of PAHs in the waters of Shitou Koumen Reservoir, carry out human health risk assessment with HQ method as well as ecological risk assessment based on RQ approach and identify possible sources of 16 PAHs according to the ratios of selected PAHs.

## 2. Experimental Section

### 2.1. Study Area and Sample Collection

**Figure 1:**
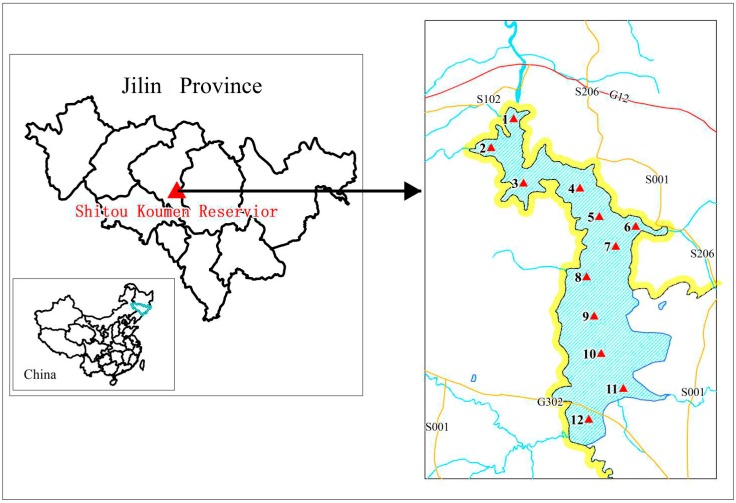
The locations of the Shitou Koumen Reservoir and the sampling sites.

Located in Jilin Province of China, the Shitou Koumen Reservoir (43°58′ N, 125°45′ E) is the primary water source for eight million local residents and it is also utilized in the irrigation of farmlands as well as in fish farming. A constructed wetland is located in the water inlet of the reservoir for polluted river water treatment. In August 2014 (wet season), a total of 12 surface water samples (0.5–1 m) covering all directions were collected from the Shitou Koumen Reservoir. Three subsamples were mixed thoroughly for the purpose of obtaining a composite sample for each site. The distance between every two subsamples was around 50 m and the sampling locations are shown in Figure 1. The following several points have to be noted: (1) water outlet, referring to the reservoir water flowing into the downstream river and water inlet, referring to the river water flowing into the reservoir, are shown in two sites (S1 and S12); (2) fish-culturing area was represented by a site, S10; (3) two sites (S11 and S12) represented the wetland area; and (4) main potential polluted tributaries were represented by three sites (S2, S6 and S12). Meanwhile, 10 liters of water were collected from each sampling site, transported to the laboratory immediately and analyzed within 24 h. 

### 2.2. Chemical Analysis

The concentrations of 16 PAHs recommended as priority pollutants by the United States Environmental Protection Agency (USEPA) were measured: naphthalene (Nap), acenaphthene (Ace), acenaphthylene (Acy), fluorene (Flo), phenanthrene (Phe), anthracene (Ant), fluoranthene (Fla), pyrene (Pyr), benz(a)anthracene (BaA), chrysene (Chr), benzo(b)fluoranthene (BbF), benzo(k)fluoranthene (BkF), benzo(a)pyrene (BaP), dibenz(a,h)anthracene (DahA), indeno(1,2,3-cd)pyrene (IcdP) and benzo(g,h,i)perylene (BghiP). Mixed standard solution of 16 priority PAHs (100 μg/mL in dichloromethane for each) was purchased from the National Standard Material Center (Beijing, China) and all solvents used were of chromatographic grade. 

With regard to the extraction of water samples, the published procedure by Wang was followed [1]: firstly, the water samples were filtered with 0.45 μm glass fibre filters and then the filtered water samples were extracted with a solid phase extraction (SPE) system (Cleanert S C18-SPE, Agela Technologies Inc., Tianjin, China). The SPE cartridges were prewashed with 5 mL of dichloromethane, 5 mL of methanol as well as 5 mL of deionized water with a constant rate of 6 mL/min successively. Two liters of filtered water samples passed through the prewashed cartridges at a constant rate of 6 mL/min. The cartridges were eluted with 10 mL of dichloromethane, which were then concentrated to about 1mL with a rotary evaporator (RE-52AA, Shanghai Yarong Inc., Shanghai, China). 

Sixteen PAHs were quantified with a gas chromatography (GC) equipped with a flame ionization detector (Clarus 680, PerkinElmer Inc., Waltham, MA, USA). The detector was set at a temperature of 300 °C and a HP-5 capillary column (30 m × 0.25 mm i.d. with a 0.25-μm film thickness) was used to separate target analytes. In addition, the carrier gas was helium (99.999%) and set at 1 mL/min. Then 1 μm treated water sample was splitless injected under 250 °C. After initially setting the column temperature at 80 °C for 1 min, the temperature was increased to 255 °C at 15 °C/min for 1 min. After that, it was raised to 265 °C at a rate of 2.5 °C/min for 1 min and finally it was increased to 300 °C at 2.5 °C/min for 5 min.

### 2.3. Quality Controls

All data were subjected to strict quality insurance and blank along with duplicate sample analyses were carried out. The method of external standards was also applied to the quantification of PAHs and the correlation coefficients for calibration curves were all higher than 0.998. In order to analyze the recovery rate before pretreatment of water samples and quantified analysis, six water samples were spiked mixed standard solutions. The recovery rates for 16 PAHs were 75%–105% and the relative standard deviations (RSDs) ranged from 4.2% to 13.6%. Under the analytical condition of this study, the detection limits for 16 PAHs were 0.56–4.21 ng/L.

### 2.4. Calculation of Risk Quotients

When it comes to the evaluation of the potential risk of a certain pollutant or complex pollutants to aquatic ecosystem [10], the risk quotient (RQ) can be used and the RQ is calculated by Equation (1):

RQ = (Monitoring concentration of i) / (Allowable concentration of i)
(1)

where allowable concentrations of pollutants are recommended by USEPA (2008) [13]. 

The risk can be evaluated more accurately with two methods and the RQs under the best and worst cases are calculated as follows:

RQ(wc) = (Maximum monitoring concentration of i)/(Allowable concentration of i)
(2)

RQ(bc) = (Minimum monitoring concentration of i)/(Allowable concentration of i)
(3)

When the RQ (wc) for a certain pollutant is less than 1, the potential adverse effects caused by the pollutant exposure are the minimum; whereas if the RQ (bc) for a certain pollutant is greater than 1, the potential adverse effects caused by the pollutant exposure may be severe. More refined analysis can be provided by the methods of best-case RQ and worst-case RQ for the evaluation of the risk levels which can usually be identified by RQ (bc) > 1 or RQ (wc) < 1 independently [10,11]. 

### 2.5. Human Risk Assessment

The channels of pollutants in the water to human body mainly include direct ingestion as well as dermal adsorption, which are respectively calculated by Equations (4) and (5) [9,14,15]:

Di = (C × IR × EF × ED)/(BW × AT)
(4)

Dd = (C × SA × Kp × ET × EF × ED × CF)/(BW × AT)
(5)

where Di (μg/(kg day)) is the exposure dose through direct ingestion; Dd (μg/(kg day)) is the exposure dose through dermal absorption; C (μg/L) stands for the monitoring concentration of a certain pollutant; IR (L/day) means the rate of direct ingestion (1.4 L/day); EF (day/year) is the exposure frequency (365 days per year for direct ingestion and 350 days per year for dermal absorption); ED (year) represents the exposure duration equal to a lifetime (70 year); BW (kg) is the average body weight (57 kg); AT (day) is the average time for non-carcinogens and carcinogens (ED × 365 days); SA (cm^2^) is the exposed skin area (18,000 cm^2^); Kp (cm/h) refers to the dermal permeability coefficient; ET (h/day) means the exposure time of shower and bathing (0.25 h/day); and CF (L/1000 cm) stands for the unit conversion factor [14]. The variables of IR, EF, ED, SA, BW and ET, the sources and values of which were shown in Table 1, were collected from the USEPA and Ministry of Health of the People’s Republic of China (MHPRC 2007) [16].

**Table 1:** The random variables in HQ assessment.

Definition	Units	Values
Ingestion rate (IR) ^a^	L/day	1.41
Exposure frequency (EF) ^b^	day/year	365
Exposure duration (ED) ^c^	year	73.65
Body weight (BW) ^c^	kg	53.6
Surface area (SA) ^a^	cm^2^	20,091
Exposure time during bathing and shower (ET) ^c^	min/day	20
Gastrointestinal absorption factor (ABSg) ^b^	unitless	0.5

^a^ (USEPA 1997); ^b^ (USEPA 1989); ^c^ (MHPRC 2007).

The human health risk caused by the exposure of pollutants can be classified into non-carcinogenic risk and carcinogenic risk [17]. The hazard quotient (HQ) for non-carcinogenic risk is calculated by Equation (6):

HQ = D/RfD
(6)

where D is the exposure dose calculated by Equations (4) and (5) and RfD (μg/(kg·day)) is the reference dose of a certain pollutant for non-carcinogenic risk. The ingestion reference doses (RfDi) were collected from the USEPA (2008) [13], while the dermal absorption reference doses (RfDd) are calculated by Equation (7):

RfDd = RfDi × ABSg
(7)

where ABSg refers to a factor of gastrointestinal absorption (USEPA 2004) [15].

The total non-carcinogenic risk is evaluated by the sum of HQ of each pollutant. The hazard index (HI) is calculated by Equation (8) [14]:

HI = HQ_1_+ HQ_2_ + HQ_3_ + … + HQ_n_ (8)

Lifetime carcinogenic risk (LCR) can be evaluated through the channels of direct ingestion and dermal adsorption and calculated as Equation (9) below [14]:

LCR = D × SF
(9)

where LCR refers to the probability of developing cancer over a lifetime; D (μg/(kg·day)) is the exposure dose calculated by Equations (4) and (5); and SF (μg/(kg·day)) is the carcinogenic slope factor of each pollutant, which was collected from the USEPA (1997) data [18]. Besides, the ingestion slope factor (SFi) was collected from the USEPA (2008) data [13] and the dermal absorption slope factor (SFd) is calculated by Equation (10):

SFd = SFi/ABSg
(10)

The total potential carcinogenic risk can be assessed by the risk index (RI), which is calculated by summing the LCR of each pollutant as follows [14]:

RI = LCR_1_ + LCR_2_ + LCR_3_ + … + LCR_n_ (11)

### 2.6. Statistics Analyses

As for the statistical analyses, SPSS 16.0 (IBM Co., Armonk, NY, USA) and Origin 8.0 (Origin Inc., Cleveland, OH, USA) were applied. The possible pollution sources can be determined by diagnostic ratios of selected PAHs because they are diluted to a similar extent and equally distribute in different environmental compartments [7].

## 3. Results 

### 3.1. Occurrence and Spatial Distribution of PAHs in the Water 

The concentration levels of 16 PAHs in the water of the Shitou Koumen Reservoir are presented in Table 2. All PAHs, except for DahA, were detected in the water samples. Fla boasted the highest average concentration, which was 5.66 × 10^−1^ μg/L, whereas DahA was undetected in any water samples was the lowest and the coefficients of variation (C.V) for detectable PAHs ranged from 36% to 187%.

In order to further describe the occurrence of PAHs in the water, compositional patterns of PAHs were studied. Based on the number of aromatic rings, the compositional patterns of PAHs can be classified into light (2–3 ring), 4-ring and heavy (5–6 ring) PAHs [19]. According to the findings, the concentration of 4-ring PAHs, which accounted for 56%, was the highest in water samples, the concentration of light PAHs ranked second with the proportion of 38% and that of the heavy PAHs was the lowest with 5% for 5-ring PAHs and 1% for 6-ring PAHs. 

**Table 2:** The concentration levels of 16 PAHs in the water.

PAHs (μg/L)	Minimum	Mean	Maximum	C.V % ^a^
Nap	8.10 × 10^−3^	5.10 × 10^−2^	8.50 × 10^−2^	49
Ace	ND	1.68 × 10^−1^	6.96 × 10^−1^	152
Acy	ND	2.13 × 10^−2^	3.47 × 10^−3^	64
Flo	ND	7.35 × 10^−2^	1.96 × 10^−1^	94
Phe	ND	2.02 × 10^−3^	4.75 × 10^−3^	95
Ant	1.20 × 10^−1^	5.10 × 10^−1^	7.60 × 10^−1^	41
Fla	1.02 × 10^−1^	5.66 × 10^−1^	8.33 × 10^−1^	36
Pyr	ND	2.18 × 10^−1^	4.11 × 10^−1^	70
BaA	ND	7.80 × 10^−4^	3.70 × 10^−3^	164
Chr	ND	4.01 × 10^−1^	9.27 × 10^−1^	71
BbF	ND	1.93 × 10^−3^	7.19 × 10^−3^	142
BkF	ND	9.40 × 10^−2^	2.22 × 10^−1^	77
BaP	ND	5.78 × 10^−4^	2.90 × 10^−3^	187
DahA	ND	-	ND	-
IcdP	ND	3.21 × 10^−3^	1.39 × 10^−2^	155
BghiP	ND	1.68 × 10^−2^	4.98 × 10^−2^	120

**^a^** Coefficients of variation. ND Not detected. - No data.

**Figure 2:**
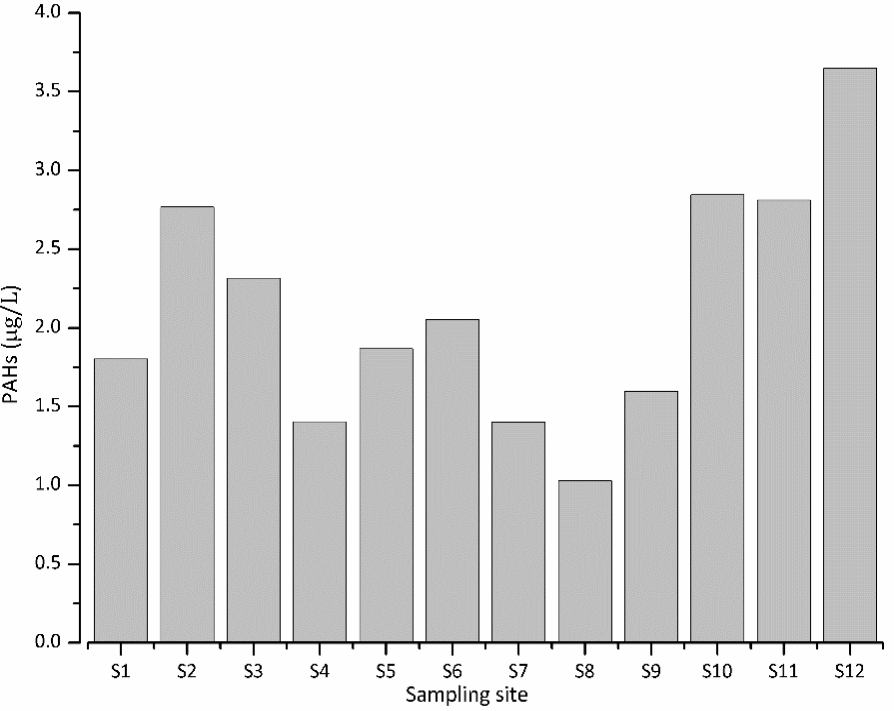
The concentration levels of total PAHs in the water.

The spatial variation of total 16 PAHs in the water are shown in Figure 2, from which it could be seen that the concentrations of total 16 PAHs at different sampling sites ranged from 1 μg/L to 3.75 μg/L. 

### 3.2. Ecological Risk Assessment

In the assessment of potential adverse impact on aquatic ecosystem, the RQs under the best and worst cases were employed. As shown in Table 3, the values of RQs for individual PAHs and total PAHs under the best and worst cases were all less than 1.

**Table 3:** Risk quotients for 16 PAHs (μg/L) in the water.

PAHs	Minimum Concentration	Maximum Concentration	RQ(bc) < 1	RQ(bc) > 1	RQ(wc) < 1	RQ(wc) > 1
Nap	8.10 × 10^−3^	8.50 × 10^−2^	●		●	
Ace	ND	6.96 × 10^−1^	●		●	
Acy	ND	3.47 × 10^−3^	●		●	
Flo	ND	1.96 × 10^−1^	●		●	
Phe	ND	4.75 × 10^−3^	●		●	
Ant	1.20 × 10^−1^	7.60 × 10^−1^	●		●	
Fla	1.02 × 10^−1^	8.33 × 10^−1^	●		●	
Pyr	ND	4.11 × 10^−1^	●		●	
BaA	ND	3.70 × 10^−3^	●		●	
Chr	ND	9.27 × 10^−1^	●		●	
BbF	ND	7.19 × 10^−3^	●		●	
BkF	ND	2.22 × 10^−1^	●		●	
BaP	ND	2.90 × 10^−3^	●		●	
DahA	ND	ND	-		-	
IcdP	ND	1.39 × 10^−2^	●		●	
BghiP	ND	4.98 × 10^−2^	●		●	
∑PAHs	2.30 × 10^−1^	4.22E+00	●		●	

### 3.3. Human Health Risk Assessment

In the process of human health risk assessment, the reference values for some pollutants had not been defined and thus only pollutants with defined reference values were investigated in this study. As presented in Table 4, all values of HQ for the measured PAHs through ingestion and dermal adsorption were less than 1. Similarly, all calculated HI were also less than 1. 

**Table 4:** Non-carcinogenic risk of PAHs.

PAHs	Ingestion			Dermal		
	Minimum	Mean	Maximum	Minimum	Mean	Maximum
Nap	1.07 × 10^−5^	6.71 × 10^−5^	1.12 × 10^−4^	2.13 × 10^−5^	1.34 × 10^−4^	2.24 × 10^−4^
Ace	0.00 × 10^0^	7.39 × 10^−5^	3.05 × 10^−4^	-	-	-
Flo	0.00 × 10^0^	4.83 × 10^−5^	1.29 × 10^−4^	-	-	-
Ant	1.05 × 10^−5^	4.47 × 10^−5^	6.66 × 10^−5^	-	-	-
Fla	6.71 × 10^−5^	3.72 × 10^−4^	5.48 × 10^−4^	1.34 × 10^−4^	7.45 × 10^−4^	1.10 × 10^−3^
Pyr	0.00 × 10^0^	1.92 × 10^−4^	3.60 × 10^−4^	-	-	-
HI = ∑HQ	1.73 × 10^−4^	7.98 × 10^−4^	1.41 × 10^−3^	2.55 × 10^−4^	8.78 × 10^−4^	1.22 × 10^−3^

LCR was used to evaluate the probability of developing cancer caused by the exposure of pollutants during a lifetime [20]. As the reference values of SFs for some pollutants had not been defined, the pollutants without defined reference values of SFs were not studied. According to Table 5, the values of LCR for both ingestion and dermal adsorption were all less than 1.00 × 10^−4^ except for Chr, whereas the average values of RI for both ingestion and dermal adsorption were all higher than 1.00 × 10^−4^ (Table 5).

**Table 5:** Carcinogenic risk of PAHs.

PAHs	Ingestion			Dermal					
	Minimum	Mean	Maximum	Minimum		Mean		Maximum	
BaA	ND	2.81 × 10^−8^	1.33 × 10^−7^	ND	5.40 × 10^−8^		2.56 × 10^−7^		
Chr	ND	1.44 × 10^−3^	3.34 × 10^−3^	ND	2.78 × 10^−3^		6.43 × 10^−3^		
BbF	ND	6.96 × 10^−8^	2.59 × 10^−7^	ND	1.99 × 10^−7^		7.38 × 10^−7^		
BkF	ND	3.39 × 10^−5^	8.00 × 10^−5^	-	-		-		
BaP	ND	2.09 × 10^−9^	1.05 × 10^−8^	ND	5.93 × 10^−9^		2.98 × 10^−8^		
IcdP	ND	1.61 × 10^−7^	5.02 × 10^−7^	ND	5.23 × 10^−7^		2.27 × 10^−6^		
RI = ∑LCR	-	1.78 × 10^−3^	3.84 × 10^−3^	-	2.79 × 10^−3^		6.44 × 10^−3^		

### 3.4. Identification of PAH Sources

**Figure 3:**
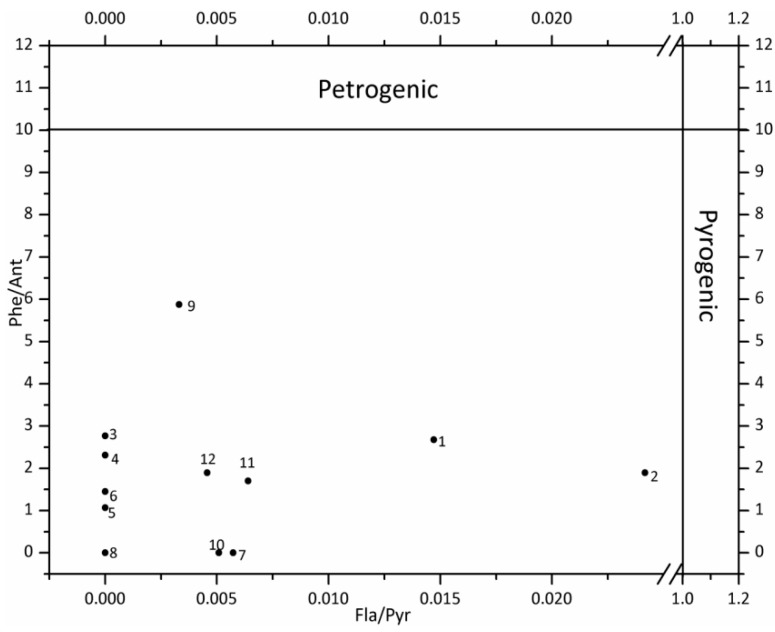
The cross-plots of 16 PAHs for the ratios of Phe/Ant *vs.* Fla/Pyr.

**Figure 4:**
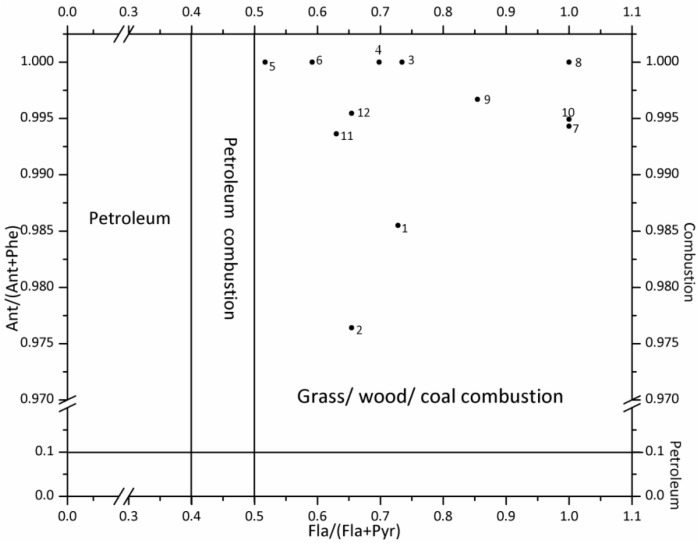
The cross-plots of 16 PAHs for the ratios of Ant/(Ant + Phe) *vs.* Flu/(Flu + Pyr).

The anthropogenic sources of PAHs include pyrogenic as well as petrogenic sources [1]. Pyrogenic sources are derived from the incomplete combustion of organic compounds such as the burning of biomass as well as fossil fuels (coal burning and vehicle emissions); petrogenic sources are derived from unburned fossil fuels, which includes oil spills as well as incineration of petroleum products (coal and crude oil) [21,22]. The pyrogenic and petrogenic sources of PAHs can be differentiated by the ratios of Phe/Ant and Fla/Pyr [23]. In order to further distinguish the PAH sources, the ratios of Ant/(Ant + Phe) and Fla/(Fla + Pyr) were adopted. The ratios of Ant/(Ant + Phe) can be used for distinguishing between combustion and petroleum sources, while the ratios of Fla/(Fla + Pyr) can be applied to differentiating the possible PAH sources of petroleum input, petroleum combustion (vehicle and crude oil) and combustion of grass, wood and coal [24]. The results for possible sources of PAHs in the water from Shitou Koumen Reservoir were shown in Figure 3 and Figure 4.

## 4. Discussion

### 4.1. Occurrence and Spatial Distribution of PAHs in the Water

It could be known from the large C.V. that the occurrence of PAHs in the water might be related to some extent to anthropogenic activities. The enrichment of light PAHs in the water can be explained by their high vapor pressure and water solubility, while the low concentration of heavy PAHs can be attributed to their lower water solubility and great tendency to adsorb onto solid phases [25]. Generally speaking, the composition of PAHs is characterized by the greater number of aromatic rings with high carcinogenicity [26]. In spite of this, it was still not sufficient to evaluate the carcinogenic risk by this method, hence the application of LCR to the further evaluation of the carcinogenic risk.

The highest concentration of total 16 PAHs was found at S12 (the water-inlet and constructed-wetland area), which might be attributed to the polluted inflowing river. The second highest concentrations occurred at S2, S10 and S11, the PAH pollution of which might be caused by polluted tributaries, fish-farming activities and the wastewater treatment process in the constructed wetland, respectively. 

### 4.2. Ecological Risk Assessment

The values of RQs for individual PAHs and total PAHs under the best and worst cases were all less than 1. Thus, both individual PAHs and complex PAHs might have little or no adverse effects on aquatic ecosystem. The potential adverse effects on aquatic ecosystem caused by the toxicity of 16 PAHs have been known, the effects on the changes of metabolism in non-target organism or toxicity by synergy have not been studied yet [27].

### 4.3. Human Health Risk Assessment

HQ was applied to the evaluation of non-carcinogenic health risk through ingestion and dermal adsorption. HQ < 1 indicates that little or no significant potential negative influences are exerted on human health, whereas potential negative influences on human health may be assumed if HQ > 1 [28]. According to the results, all HQ values for the measured PAHs through both ingestion and dermal adsorption were less than 1, which indicated little or no potential adverse effects of measured PAHs on local residents’ health through ingestion and dermal adsorption. The exposure of complex pollutants may cause interactive and/or additive effects on human health, while the total non-carcinogenic health risk from complex pollutants can be evaluated by HI [29]. If HI < 1, it means that little or no significant potential adverse effects are exerted by complex pollutants on human health, whereas the complex pollutants may cause potential adverse effects if HI > 1. The result in this study showed that the calculated HIs were all less than 1; thus, the exposure of complex PAHs had little or no potential adverse effects on the local consumers.

According to the regulatory regimes, the acceptable values of LCR are in the range of 1.00 × 10^−6^–1.00 × 10^−4^. LCR > 1.00 × 10^−4^ indicates that local residents may be faced with a potential carcinogenic risk due to the exposure of pollutants [27]. The values of LCR through both ingestion and dermal adsorption were all less than 1.00 × 10^−4^ except for Chr, suggesting that potential carcinogenic risks of the exposure of individual PAHs were low. RI was adopted in the evaluation of potential carcinogenic risks caused by synthetic effects of complex pollutants through ingestion and dermal adsorption. As a result, the average values of RI for both ingestion and dermal adsorption were all greater than 1.00 × 10^−4^, indicating a potential carcinogenic risk of the complex PAHs in the water of the Shitou Koumen Reservoir on local residents. It should also be noted that PAHs with no reference values might also have potential adverse effects on local residents.

### 4.4. Identification of PAH Sources

As shown in Figure 3, all sampling sites were exposed to a mixture pattern of pyrogenic and petrogenic sources. Based on Figure 4, the dominant source of PAHs was combustion, namely combustion of grass, wood and coal [23].

Generally, the findings of this work proved that both petrogenic and pyrogenic inputs contributed to PAH contamination in the waters of the Shitou Koumen Reservoir. The petrogenic PAHs were probably generated from oil spills caused by oil-related activities in oil refineries as well as motorboats of travelers. The vehicular emissions were possibly originated from heavy traffic on expressways surrounding the reservoir (in Figure 1). The pollution caused by vehicular emissions could be explained as follows: the atmospheric particles containing PAHs from vehicular emissions were probably transported by air masses during the monsoon season [30], and then entered into the water by atmospheric deposition. Based on the reality, burning agricultural wastes by local farmers might result in the combustion of biomass and industrial plants located in cities nearby the reservoir might lead to the coal combustion. 

## 5. Conclusions

Sixteen polycyclic aromatic hydrocarbons (PAHs) were identified in 12 water samples from Shitou Koumen Reservoir, China and all individual PAHs, except DahA, were detected in water samples. The highest and lowest concentrations of PAH in water samples were Fla (5.66 × 10^−1^ μg/L) and DahA (undetected) respectively. In addition, the highest concentration of PAH compositional patterns was for light and 4-ring PAHs which accounted for 94%. The highest concentrations of total 16 PAHs were found in constructed-wetland and fish-farming areas, indicating that fish-farming activities and wastewater treatment processes had a contribution to the enrichment of PAHs in water. According to the results of RQs, little or no adverse effects were exerted by individual and complex PAHs in the water on aquatic ecosystem of the reservoir. Similarly, little or no negative effects of individual and complex PAHs were shown by the results of HQs for non-carcinogenic and carcinogenic risk on the local comsumers. Nevertheless, a potential carcinogenic risk caused by the exposure of Chr and complex PAHs in water might be faced by local residents. Finally, it could be seen from the results of diagnostic ratios of selected PAHs that possible sources of PAHs in water were identified as oil-related activities, vehicular emissions as well as combustion of agricultural wastes and coal.
